# Supplementary material for: Impact of nephrotoxins and oxidants on survival and transport function of hiPSC-derived renal proximal tubular cells
Source: Arch Toxicol. 2025 Mar 22;99(6):2549–63. doi: 10.1007/s00204-025-04015-1 (PMC12185579; doi:10.1007/s00204-025-04015-1)
Supplement: Supplementary file 1 — Supplementary file1 (PDF 116 KB) [file 204_2025_4015_MOESM1_ESM.pdf]

Supplementary Table S1. Primer sequences used for real-time PCR.

ACTB = β-actin, AQP1 = aquaporin-1, CAD16 = cadherin 16, CAT = Catalase, CD13 = alanyl aminopeptidase, CUBN = cubilin, , β-GAL = beta galactosidase, GLUT5 = glucose transporter 5, GPX1 = glutathione peroxidase 1, GSS = glutathione synthetase, HMOX1 = Heme oxygenase 1, IL-8 = Interleukin 8, MEG = megalin, MnSOD2 = manganese-dependent superoxide dismutase, NANOG = homeobox protein, N-CAD = N-cadherin, NOS2/3 = Nitric oxide synthase 2/3, NOX1/4 = NADPH oxidase 1/4, NQO1, quinone oxidoreductase 1, NRF2 = Nuclear factor erythroid 2-related factor 2, OAT1/3 = organic anion transporter 1/3, OCT3/4 = octamer-binding transcription factor 3/4, p21 = cyclin-dependent kinase inhibitor 1, p47 = p47phox, a subunit of NADPH oxidase, PEPT1/2 = peptide transporter 1/2, RPL32 = ribosomal protein L32, SOD1 = superoxide dismutase 1, TRX = Thioredoxin, TXNRD1 = Thioredoxin reductase

Gene	Forward	Reverse
**ACTB**	GAGCACAGAGCCTCGCC	TCATCATCCATGGTGAGCTGG
**AQP1**	CATCCTCTCAGGCATCACCTC	CACACCATCAGCCAGGTCATTG
**CAD16**	AGCACGTGTGAAGTCGAAGT	ACTGAGGTTCTGGGAAGTGATG
**CAT**	CAAAATGCTTCAGGGCCGC	GAGCACGGTAGGGACAGTTC
**CD13**	TGGCCACTACACAGATGCAG	CTGGGACCTTTGGGAAGCAT
**CUBN**	TAGCTTCGTGAAGGTGTGGG	GACTGGAAGACGGCAGTGAA
**β-GAL**	TGCGCAATGCCACCCA	CAGGGCACATACGTCTGGAT
**GCLC**	ACGGAGGAACAATGTCCGAG	CAGGACAGCCTAATCTGGGAA
**GLUT5**	GCCAAAGTGCACCCAGAATG	GTCAGCCTCCCTTCCTTCAT
**GPX1**	CCGGGACTACACCCAGATGA	TTGGCGTTCTCCTGATGCC
**GSS**	ATAGCTGCTGGCCGAAAACT	TCCGTGAGTCCCACTGTC
**HMOX1**	CTGCTCAACATCCAGCTCTTTG	CTTGGTGTCATGGGTCAGCA
**IL-8**	TTGGCAGCCTTCCTGATTTCT	GGGTGGAAAGGTTTGGAGTATG
**MEG**	GCCAGTGGCCAAGAATGTGA	TCCGCGTCATCTGAACAGTC
**MnSOD2**	GCTTTCTCGTCTTCAGCACC	AGATACCCCAAAACCGGAGC

**NANOG**	ACCTCAGCTACAAACAGGTGAA	AAAGGCTGGGGTAGGTAGGT
**N-CAD**	AGGCTTCTGGTGAAATCGCA	GCAGTTGCTAAACTTCACATTGAG
**NOS2**	CTCCACATTGTTGTTGAT	AATCCAGATAAGTGACATAAG
**NOS3**	TGGAGTCTTGTGTAGGATA	CAAGGAGACGAAGAGAAC
**NOX1**	AATGTCACATACTCCACTG	CTCTCCAGCCTATCTCAT
**NOX 4**	TGACAGGTTTGTTGTCCTG	CTGGAAGAACCCAAGTTCCA
**NQO1**	ACCTTGTGATATTCCAGTTCCCC	GAACACTCGCTCAAACCAGC
**NRF2**	AGTGACTGAAACGTAGCCGA	CAGCTTTTGGCGCAGACATT
**OAT1**	AGTATGGAGGTACTCCGGGC	GCATGGAGAGGCAGAGGAAG
**OAT3**	CTTTGTGCCCTTGGACTTGC	GGAAGAGGCAGCTGAAGGAG
**OCT3/4**	ACCCACACTGCAGCAGATCA	CCACACTCGGACCACATCCT
**p21**	AGTCAGTTCCTTGTGGAGCC	GACATGGCGCCTCCTCTG
**p47**	GGGCGCGGATTTATAGCAGT	CCCCAGTCACTCACGTTTCC
**PEPT1**	CAAGTGCATCGGTTTTGCCA	CTCTTTAGCCCAGTCCAGCC
**PEPT2**	CTGGGAGGACAAGTGGTACA	AGTCCGTTCCTCTGCATGTT
**RPL32**	GTTACGACCCATCAGCCCTTG	CATGATGCCGAGAAGGAGATGG
**SOD1**	GCCTCATAATAAGTGCCATA	TCTGTTTCAATGACCTGTATT
**TRX**	GATGGTCAAGAGCCCAACCA	CCGGGAAGTATCTCGGTGTG
**TXNRD1**	AGCATGTCATGTGAGGACGG	CCAATTCCGAGAGCGTTCCT
